# Glutamine coated titanium for synergistic sonodynamic and photothermal on tumor therapy upon targeted delivery

**DOI:** 10.3389/fbioe.2023.1139426

**Published:** 2023-04-10

**Authors:** Lina Zhang, Pengfeng Zhu, Ting Wan, Huaiyan Wang, Zhilei Mao

**Affiliations:** ^1^ Changzhou Maternal and Child Healthcare Hospital, Changzhou Medical Center, Nanjing Medical University, Changzhou, China; ^2^ State Key Laboratory of Reproductive Medicine, Center for Global Health, Nanjing Medical University, Nanjing, China; ^3^ Key Laboratory of Modern Toxicology of Ministry of Education, School of Public Health, Nanjing Medical University, Nanjing, China

**Keywords:** TiO_2−x_@GL, sonodynamic (SDT), photothermal (PTT), synergistic tumor therapy, glutamine addiction

## Abstract

**Introduction:** The application of titanium dioxide nanoparticles (TiO_2_ NPs) for cancer therapy has been studied for decades; however, the targeted delivery of TiO_2_ NPs to tumor tissues is challenging, and its efficiency needs to be improved.

**Method:** In this study, we designed an oxygen-deficient TiO_2-x_ coated with glutamine layer for targeted delivery, as well as the enhanced separation of electrons (e-) and holes (h+) following the joint application of sonodynamic therapy (SDT) and photothermal therapy (PTT).

**Results:** This oxygen-deficient TiO_2-x_ possesses relatively high photothermal and sonodynamic efficiency at the 1064 nm NIR-II bio-window. The GL-dependent design eased the penetration of the TiO_2-x_ into the tumor tissues (approximately three-fold). The *in vitro* and *in vivo* tests showed that the SDT/PTT-based synergistic treatment achieved more optimized therapeutic effects than the sole use of either SDT or PTT.

**Conclusion:** Our study provided a safety targeted delivery strategy, and enhanced the therapeutic efficiency of SDT/PTT synergistic treatment.

## Introduction

Owing to its remarkable biocompatibility, high photoactivity, excellent stability, and low toxicity, titanium dioxide nanoparticles (TiO_2_ NPs) were regarded as a promising anticancer material and were studied in-depth (Cesmeli and Biray Avci, 2019; Ziental et al., 2020). Being a typical emerging photochemical sensitizer, TiO_2_ NPs were highly effective in tumor therapy, and generated reactive oxygen species (ROS) in numerous types of cancer cells resulting in cell death upon illumination with light, majorly ultraviolet (UV) light (Fujiwara et al., 2015; Lee et al., 2018). In addition, TiO_2_ NPs were considered to be a promising acoustic sensitizer in tumor therapy owing to their remarkable stability compared to that of the organic acoustic sensitizer (Li et al., 2021). However, the ROS production of TiO_2_ NPs is relatively low because of the rapid recombination of their electron (e-) and hole (h+) (Dai et al., 2022). Therefore, the application of TiO_2_ NPs as a photochemical or acoustic sensitizer would necessitate prolonged illumination of UV light and yield low efficiency in ROS production (Gao et al., 2019; Kim et al., 2022). To resolve this challenge, researchers reformed the physicochemical features of TiO_2_ NPs to realize easier ROS release near the infrared-II biowindow, and proposed a SDT (sonodynamic therapy)/PTT (photothermal therapy)-based synergistic strategy for cancer therapy (Han et al., 2018).

SDT/PTT-based synergistic cancer therapy is a promising strategy, particularly considering TiO_2_ NPs, which respond to both sound and light simultaneously (Behnam et al., 2018; Chu et al., 2019). Therefore, SDT/PTT synergistic therapy can shorten the illumination duration for activation, thus compensating for the deficiency in ROS generation. The accumulation of sufficient nanoparticles in the tumor tissues is an essential premise for all nanomedicne treatments, therefore, the target delivery of TiO_2_ NPs to the cancer cells should be enhanced to the extent possible to improve efficacy and prevent adverse side effects (Kim et al., 2020; Liang et al., 2021). Therefore, a modified delivery method should be proposed to achieve this goal.

A previous study suggested that most of the cancer cells exhibited the phenomenon of glutamine (GL) addiction, that is, the tumor cells actively absorb and accumulate GL in tumor tissues for growth (Altman et al., 2016). Although *in vitro* research indicated that GL stimulated cancer cell growth (Knox et al., 1969), certain studies indicated that the GL supplementation did not stimulate tumor tissue growth in tumor-bearing mice models (Klimberg et al., 1996). Therefore, this unique feature of tumor cells provided a potential and safe strategy for the targeted delivery of TiO_2_ NPs.

This study aimed to synthesize the TiO_2-x_@GL NPs based on the “sugar coated bullet” theory in this study. We synthesized TiO_2-x_ NPs and coated them with GL for the targeted delivery of nanoparticles to cancer cells *via* the tumor cells’ active uptake of GL, thus increasing the accumulation of TiO_2-x_@GL in tumor tissues, which consequently meant to treat the breast cancer, a common and severe cancer disease that affecting women health. Therefore, the 4T1 cancer cells and the tumor-bearing nude-mice mice were applied for the functional verification.

## Methods

### Synthesis of TiO_2-x_@GL NPs

TiO_2-x_ NPs were prepared through a an aluminum (Al) reduction method reported in a previous study (Wang et al., 2013; Han et al., 2018). To improve the tumor-targeting capability of TiO_2-x_ NPs, GL (#J61560, Thermos Scientific) was harnessed to modify the surface of TiO_2-x_ NPs. Categorically, 50 mg GL was added to TiO_2-x_ NPs solution (1 mg/ml, 10 ml) and then subjected to 4-h sonication in an ice bath. The resulting TiO_2-x_@GL NPs were acquired through centrifugation (10000 r/min, 10 min) three times and washing with deionized water.

### Characterization of TiO_2-x_@GL NPs

Transmission electron microscopy (TEM) was used to observe the morphology of TiO_2-x_ and TiO_2-x_@GL; X-ray diffraction (XRD) patterns were obtained using the Rigaku D/MAX-2200 PX XRD system; and the parameters were set as Cu Kα, 40 mA, and 40 kV. Elements of Ti, O, and N were detected by sectional energy-dispersive spectroscopy (EDS) with corresponding color mapping. The size distribution and ζ potential measurements of the particles were conducted on a Zetasizer system (Nano ZS90, Malvern Instrument Ltd.). The UV-vis-NIR absorption spectrum was used to record *via* Shimadzu UV-3600 UV-vis-NIR spectrometer. The irradiation source for photothermal hyperthermia was the 1064 nm multimode pump laser (Shanghai Connect Fiber Optics Co. Ltd.), and the ultrasound irradiation for sonodynamic therapy was conducted using an Intelect Transport Ultrasound (Chattanooga Group, United States). The Agilent 725 inductively coupled plasma-optical emission spectrometer (Agilent Technologies) was used to confirm the quantitative analysis of the contents of nanoparticles. The intracellular uptake of nanoparticles and cell apoptosis levels were detected by flow cytometry (Becton, Dickinson and Company, United States), and confocal laser scanning microscopy images were recorded using FV1000 (Olympus Company, Japan).

### Cell culture

The 4T1 cells were purchased from the American Type Culture Collection: The Global Bioresource Center (#CRL-2539). The cells were cultured with RPMI-1640 medium, supplemented with 10% fetal bovine serum (FBS) (#10100147C, GIBICO), in a 5% CO_2_ atmosphere at 37°C. The cell culture medium was replaced daily and the cells were treated upon reaching 60% confluence.

### *In vitro* sonodynamic and photothermal treatment

The commercial Cell Counting Kit-8 (CCK-8) assay kit (#C0037, Beyotime Biotechnology) was used to evaluate the sonocatalytic and photothermal treatment for killing cancer cells. 4T1 cells were seeded in 96-well plates and cultured with RPMI-1640 medium containing 10% FBS for 12 h at a proper density, and then TiO_2-x_@GL (Ti concentration 50 ppm) was added for co-incubation. The cell viabilities in each group were determined by comparison with the control group. The laser power intensities were set as 1.5 W/cm2, and the US irradiation parameters as 1 MHz, 50% duty cycle, 1.0 W/cm2, and 200 s.

### Reactive oxygen species detection

4T1 cells were planted and co-incubated with TiO_2−x_@GL at 37°C for 4 h. After incubation, the medium was removed and the cells were washed three times with PBS. To measure the ROS production by flow cytometry, the DCFH- DA (#S0033S, Beyotime Biotechnology) was added and incubated for 1 h. The cells were treated by US radiation for 5 min. After different treatments, the cells were collected to determine the intracellular fluorescence intensity of DCF.

### Evaluation of *in vitro* photothermal effect of TiO_2−x_@GL

The infrared thermal image recorder (FLIR TM A325SC camera) was used to evaluate the photothermal effects of TiO_2−x_@GL by recording the temperature changes during laser irradiation in the NIR-II biowindow (1064 nm). TiO_2−x_@GL were dispersed in deionized water at different Ti concentrations (0, 6.25, 12.5, 25, 50, 100 and 300 ppm) and exposed to 1064 nm pump laser irradiation at a laser power density of 1.5 W/cm2. In addition, the temperature increase of TiO_2−x_@GL at a Ti concentration of 400 ppm as irradiated by a 1064 nm laser at different power intensities (0, 0.5, 1.0 and 1.5 W/cm2) was determined.

### Evaluation of cell migration ability

The 4T1 cells were re-suspended with serum free medium, then 100 ml of the cell suspension was added to the upper chamber of the transwell plate at a proper density, after which the chamber was placed into the well filled with a serum-containing medium. After culturing for an additional 12 h, cells were fixed with 4% paraformaldehyde (#C104188, Aladdin), and then stained with 0.1% crystalviolet (#C0121, Beyotime Biotechnology) for 15 min at room temperature (22°C). Upon erasure of the cells on the bottom of the upper chamber, the well was observed and the migrated cells were counted under the light microscope. The cell number was collected from four independent fields from different directions.

### Distinction between living and dead cells

The living and dead cells were distinguished using a commercial Calcein-AM/PI assay kit (#04511, Sigma-Aldich). The living cells were indicated with Calcein-AM (green) and the dead cells were stained red with PI. After staining, the cells were observed using a confocal laser scanning microscope (CLSM).

### Establishment of tumor-bearing model and *in vivo* synergistic cancer therapy

The animal study was approved by the animal ethnic committee of Nanjing Medical University. To establish the tumor bearing model, 4T1 cells (1 × 10^6^ cells) were suspended in 100 μl of PBS and injected into the right side of the back of mice (C57BL/6). Finally, 40 female 4T1 tumor-bearing mice were successfully established and fed at the Laboratory Animal Center. After the tumors grew to nearly 50 mm^3^, the mice were divided into 5 groups (n = 8) as follows: 1) Control group (treated with saline), 2) TiO_2−x_@GL only group, 3) TiO_2−x_@GL + laser group (injected with TiO_2−x_@GL followed by 1064 nm laser), 4) TiO_2−x_@GL + US group (treated with TiO_2−x_@GL + US irradiation), and 5) TiO_2−x_@GL + laser + US group (injected with TiO_2−x_@GL followed by laser and US irradiation). The injection dose of TiO_2−x_@GL was 15 mg/ml. The injection of 4T1 cells was recorded as day −7, and the mice received treatment on day 0. After intravenous injection of TiO_2−x_@GL for 4 h, the 1064 nm laser (1.5 W/cm^2^, 10 min) or the US irradiation (1 MHz, 50% duty cycle, and 1.0 W/cm^2^, 240 s) were administered to carry out the therapeutic plan. For the synergistic treatment, the mice received laser and US irradiation treatment on day 0, and the following US treatment was administered on days 3 and 5. The body weight and length and width of tumors were measured every 2 days using the digital scale and caliper, respectively. The tumor volume was calculated as follows: tumor volume (mm^3^) = ab^2^/2, a = the maximum length (mm), b = the minimum width (mm).

### Contribution to SDT/PTT-based synergistic cancer therapy

The inhibitory rate of cell growth was calculated using the equation: % Growth inhibition = [(1 − OD extract treated)/(OD negative control)] × 100. The percentage contribution of each element (TiO_2-x_@GL, US and NIR) to the SDT/PTT-based synergistic cancer therapy was calculated by % contribution= (% Growth inhibition/total growth inhibition) × 100.

### Biodistribution of TiO_2−x_@GL in tumor tissues

To verify that TiO_2−x_@GL could penetrate barriers and accumulate in tumor tissues, the bio-distributions of TiO_2−x_ and TiO_2−x_@GL in tumor tissues were determined in 4T1 female breast tumor bearing mice (n = 5); they were randomly divided into three groups and intravenously injected with TiO_2−x_ and TiO_2−x_@GL in saline at a dosage of 50 ppm. The mice were euthanized after 4 h of injection and dissected to collect the tumors. These tissues were weighed, homogenized, and dissolved in aqua regia. Then the Ti element content in tumor tissue was measured using Inductively Coupled Plasma Optical Emission spectroscopy (ICP-OES), and the distribution was calculated using the original dose per gram of tissue.

### Pathological changes, cell apoptosis, and cell proliferation after different treatments

Pathological changes, cell apoptosis, and cell proliferation were revealed by hematoxylin and eosin (H&E) staining (#C0105S, Beyotime Biotechnology), tunel assay (#25879, Cell Signal), and Ki-67 antibody (#9129, Cell Signal), respectively. The H&E staining was carried out according to a previous study (Yuan et al., 2022), and Tunel staining was performed using a commercially available assay kit according to the manufacturer’s instructions. Cell proliferation was revealed by immunohistochemistry with an anti-Ki-67 antibody combined with a goat-anti-rabbit secondary antibody.

### Statistical analysis

The data were presented as mean ± standard deviation (SD), and the difference between two groups was analyzed on the basis of the two-tailed t-test (*, *p* < 0.05; **, *p* < 0.01; ***, *p* < 0.001).

## Results

### Synthesis and characterization of TiO_2-x_ NPs and TiO_2-x_@GL NPs

**FIGURE 1:**
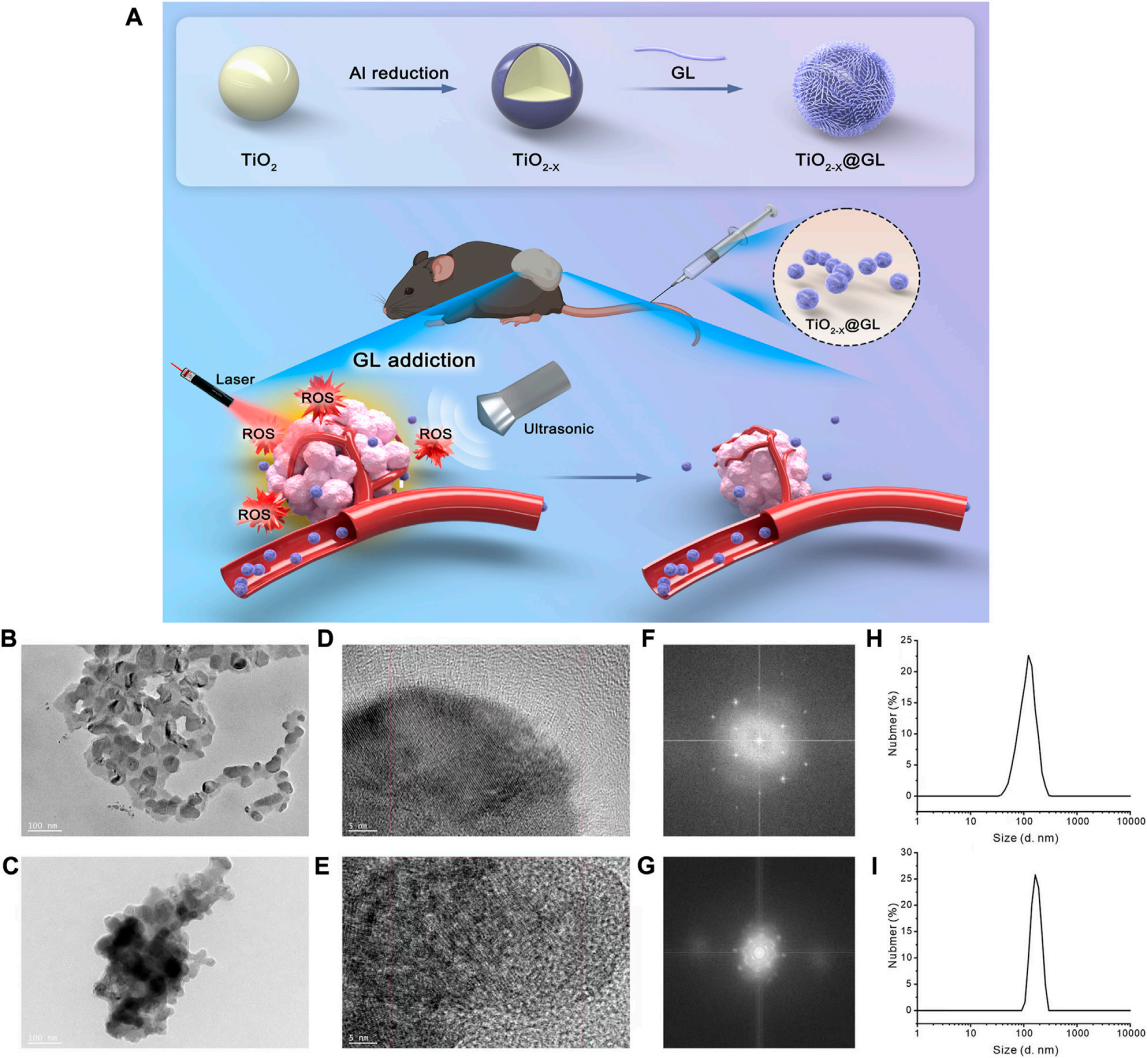
Characteristics of TiO_2-x_ NPs and TiO_2-x_@GL NPs **(A)** Graphical information of the synthesis of TiO_2-x_ NPs and the synergistic SDT/PTT cancer therapy. **(B, C)** Low-magnification TEM images of TiO_2-x_ NPs **(B)**; and TiO_2-x_@GL NPs **(C, D, E)** High-resolution TEM images of TiO_2-x_ NPs **(D)**; and TiO_2-x_@GL NPs **(E, F, G)** SAED pattern images of TiO_2_-x NPs **(F)**; and TiO_2-x_@GL NPs **(G, H, I)** DLS of TiO_2-x_ NPs **(H)**; and TiO_2-x_@GL NPs **(I)**.

**FIGURE 2:**
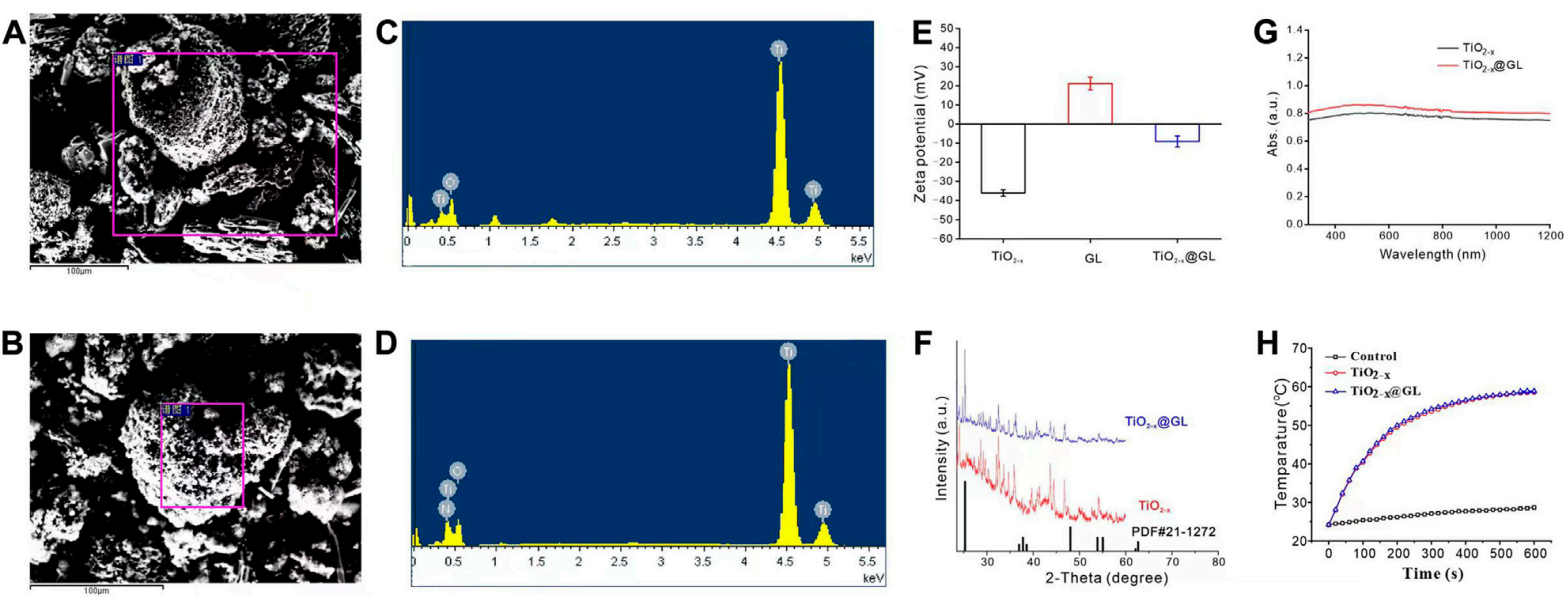
Characteristics of TiO_2-x_ NPs and TiO_2-x_@GL NPs **(A, B)** SEM images of TiO_2-x_ NPs **(B)**; and TiO_2-x_@GL NPs **(C, C, D)** EDS of TiO_2_-x NPs **(C)** obtained based on the purple rectangle area in **(A)**; and TiO_2-x_@GL NPs **(D)** obtained based on the purple rectangle area in **(B, E–G)** Zeta potentials **(E)**; XRD **(F)**; UV-Vis-NIR spectra **(G)**; photothermal heating curves **(H)** of TiO_2-x_ NPs and TiO_2-x_@GL NPs.

The synthesis progress of TiO_2-x_@GL NPs and the synergistic SDT/PTT therapy are indicated in Figure 1A. TEM were employed to identify the morphology of TiO_2-x_ NPs and TiO_2-x_@GL NPs. As indicated in Figure 1B, TiO_2-x_ displayed a uniform, spherical structure with a mean size of 50 nm. After loading into GL, there was no apparent change in the morphology of the fabricated TiO_2-x_@GL NPs compared with TiO_2-x_ NPs (Figure 1C). Based on the high-resolution TEM imaging results and corresponding SAED patterns of TiO_2-x_ NPs and TiO_2-x_@GL NPs (Figures 1D–G), the encasement of GL did not change the crystalline structure of TiO_2-x_ NPs. The DLS results indicated the surface encasement of GL increased hydrodynamic particle size of TiO_2-x_ NPs from 122.4 to 164.2 nm (Figures 1H, I). There was a remarkable elevation of nitrogen (N) in the TiO_2-x_@GL NPs group compared with the TiO_2-x_ NPs group as revealed in the SEM images (Figures 2A, B) and EDS results (Figures 2C, D), suggesting the successful loading of TiO_2-x_ NPs into GL. Despite the changes in ζ potential (Figure 2E) before and after GL encasement (from −36.1 to −9.1 mV), the crystal peaks of TiO2-x NPs and TiO_2-x_@GL NPs in XRD measurement matched the standard crystal structure of TiO_2-x_ NPs (JCPDS No. 21-1272), suggesting the structure of TiO_2-x_ NPs is well preserved after the surface encasement of GL (Figure 2F). Additionally, the UV-Vis-NIR optical absorption curves (Figure 2G) and the photothermal heating curves (Figure 2H) of TiO_2-x_ NPs and TiO_2-x_@GL NPs show that the NIR wave-absorbing capability and the photothermal performance were not compromised, and collectively demonstrated that both TiO_2-x_ NPs and TiO_2-x_@GL NPs could serve as PTT agents for tumor treatment.

### *In vitro* sonodynamic and photothermal effects of TiO_2−x_@GL

**FIGURE 3:**
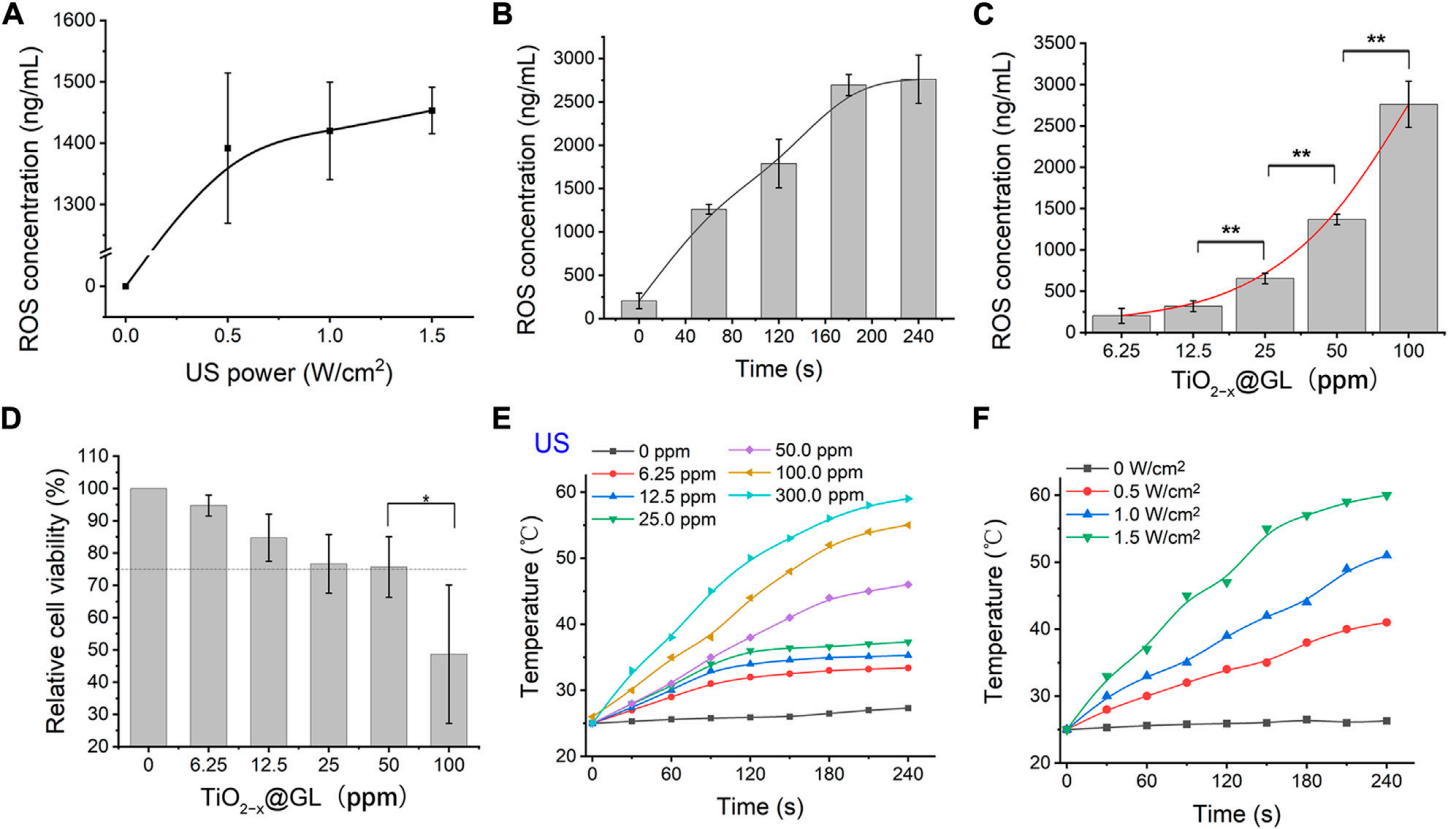
*In vitro* sonodynamic and photothermal effects of TiO_2−x_@GL. **(A)** ROS generation behavior of TiO_2−x_@GL under different ultrasonic intensities *in vitro*. **(B)** Cumulative ROS concentration curve of TiO_2−x_@GL subjected to continuous ultrasound irradiation (1.5 W/cm^2^). **(C)** Comparison of the effects of different particle concentrations on the ROS generation. **(D)** Cytotoxicities of various concentration of TiO_2−x_@GL to normal epithelial cells. **(E)** Elevated concentrations (0, 6.25, 12.5, 25, 50, 100, and 300 ppm) of TiO_2-x_@GL irradiated by NIR-II (1064 nm) at a power intensity of 1.5 W/cm^2^. **(F)** Photothermal heating curves of 50 ppm TiO_2−x_@GL irradiated at varied power densities (0, 0.5, 1.0, and 1.5 W/cm^2^).

The generation of ROS could be influenced by numerous factors, such as US irradiation, duration, and the sonosensitizer concentration. First, generation of ROS increased as ultrasound intensity increased; the ultrasound power duration was 180 s and the TiO_2-x_@GL concentration 50 ppm (Figure 3A). To determine the influence of 40-kHz ultrasound in ROS generation kinetics, we determined the cumulative ROS generation from the 50 ppm TiO_2-x_@GL (Figure 3B). Sustained INS release from TiO_2-x_@GL was achieved through ultrasound irradiation at 1.0 W/cm2. As shown in Figure 3C, the increased particle concentration treatment led to a slight increase in ROS generation 180 s after the 1.0 W/cm2 ultrasound irradiation treatment. Conversely, TiO_2-x_@GL at high concentration (>100 ppm) suppressed cell growth (<75%). Given the aforementioned results, an optimized procedure (1.0 W/cm2 of US power, 180 s, and 50 ppm TiO_2-x_@GL) was adopted to achieve high ROS generation. Furthermore, the photothermal performance of TiO_2-x_@GL aqueous solution was further evaluated at different concentrations (0, 6.25, 12.5, 25, 50, 100, and 300 ppm) and 1.5 W/cm2. The temperature could reach as high as 59.2°C (300 ppm), which is sufficient to kill cancer cells by hyperthermia. The laser power dependent photothermal effect (0, 0.5, 1.0 and 1.5 W/cm2) was also demonstrated (Figure 3F).

### *In vitro* SDT/PTT-based synergistic cancer therapy

**FIGURE 4:**
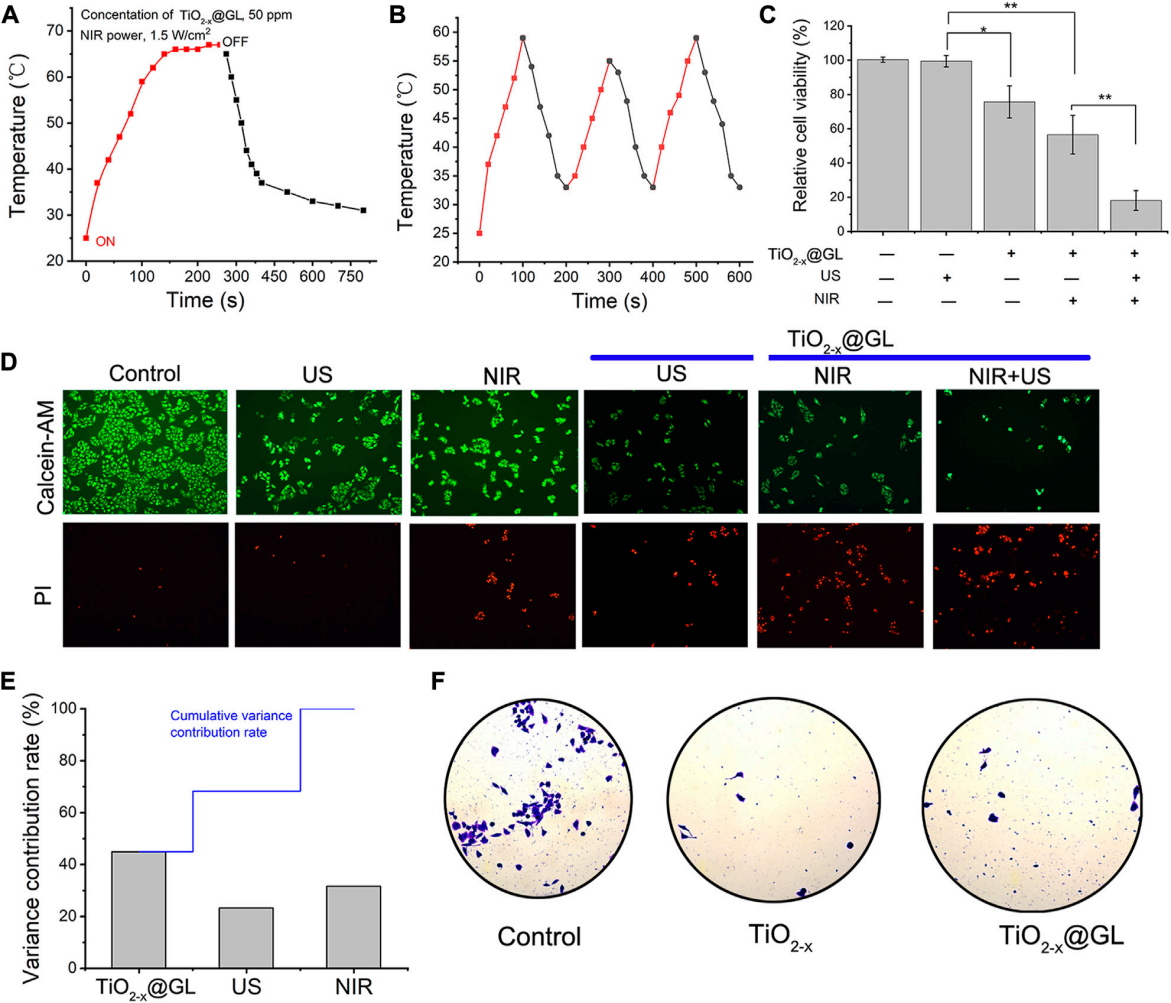
*In vitro* SDT/PTT-based synergistic cancer therapy. **(A)** Photothermal performance of the aqueous dispersion of TiO_2-x_@GL (50 ppm) under NIR irradiation at a power intensity of 1.5 W/cm^2^ for periods after US, and the temperature tended to stabilize when the laser cut off; **(B)** Heating curve of TiO_2−x_@GL dispersed in water for three cycles at a power intensity of 1.5 W/cm^2^; **(C)** Relative cell viability of 4T1 cells after different treatments, including control (without treatment), TiO_2-x_@GL only, US only, TiO_2−x_@GL combined with NIR, TiO_2-x_@GL combined with US irradiation, and TiO_2-x_@GL combined with NIR/US coirradiation. **(D)** CLSM images of 4T1 cells after different treatments, which were stained by PI (red fluorescence) and calcein-AM (green fluorescence). The scale bar is 40 μm; **(E)** Variance contribution rate of TiO_2-x_@GL, US and NIR; **(F)** Plate counting experiments of control, TiO_2-x_, and TiO_2-x_@GL.

In this study protocol, the 4T1 cells were treated by SDT and PTT (sequential treatment). Categorically, 4T1 cancer cells were incubated with TiO_2-x_@GL nanoparticles for 4 h after US irradiation and NIR-II laser. The parameters of US irradiation were set as 1 MHz, 50% duty cycle, and 1.0 W/cm^2^, 200 s. The NIR-II laser was set at a power density of 1.5 W/cm^2^. The temperature rose quickly, and reached as high as 66°C (300 ppm), which was sufficient to kill cancer cells by hyperthermia (Figure 4A). As the laser is switched off, the temperature decreased rapidly. Notably, the extremely high temperature generated an obvious ablation and smoking phenomenon under NIR-laser irradiation. There was no obvious deterioration during three laser on/off cycles for the photothermal performance of TiO_2-x_@GL nanoparticles, showing the high photothermal stability of TiO_2-x_@GL nanoagents for photothermal hyperthermia (Figure 4B). The standard CCK-8 assay was initially conducted to investigate the *in vitro* 4T1 cell killing efficacy of TiO_2-x_@GL exposed to 1064 nm laser, TiO_2-x_@GL irradiated by US activation, and TiO_2-x_@GL combined with US and 1064 nm laser irradiations. In the SDT and PTT treated group, the cancer-cell viability incubated with TiO_2-x_@GL as PTT agent for 4 h decreased with a cell-viability rate of 19.2% (Figure 4C). Furthermore, after various treatments, the cell-killing effect was directly observed by CLSM, where the live and dead cells were stained by calcein-AM (green) and PI (red), respectively. A host of dead cells were observed in the TiO_2-x_@GL combined with US and laser group, indicating that the extensive occurrence of cell apoptosis and death for synergistic SDT and PTT (Figure 4D). From the average contribution rate of inter-regional differences, the TiO_2-x_@GL had an enormous contribution rate, with an average contribution rate of 44.95%. The US and NIR variance contribution rates were 23.33% and 31.72%, respectively (Figure 4E). As reported earlier, the TiO_2_ could inhibit tumor cell migration. Transwell experiments showed that the viable cells in TiO_2-x_@GL and TiO_2-x_ groups were significantly less than those in the control group. This indicates that the TiO_2-x_@GL could inhibit tumor cell migration, like TiO_2_ (Figure 4F).

### *In vivio* SDT/PTT-based synergistic cancer therapy

**FIGURE 5:**
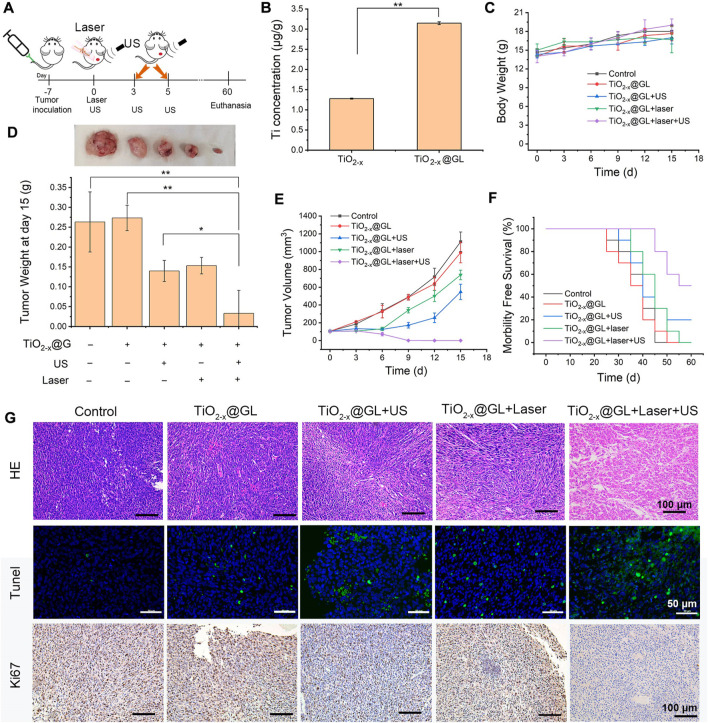
*In vivo* synergistic SDT/PTT on tumor suppression in the NIR-II biowindow. **(A)** Schematic of synergistic SDT and PTT as assisted by TiO_2-x_@GL for tumor eradication. **(B)** Accumulated Ti in the tumor after the intravenous injection of TiO_2−x_ or TiO_2−x_ @GL for 4 h. **(C)** Time-dependent body-weight curves of 4T1 tumor-bearing mice in groups of control group, TiO_2-x_@GL group, TiO_2−x_@GL combined with US group, TiO_2−x_@GL combined with laser group and TiO_2−x_@GL combined with the laser and US therapy group. **(D)** Tumor weight of different groups on day 15. **(E)** Time-dependent tumor-volume curves during the 15 days. **(F)** Survival curves of 4T1 tumor-bearing mice after different treatments. **(G)** H&E staining, Tunel staining, and Antigen Ki-67 immunohistochemistry staining in tumor region of each group after the treatments. *p < 0.05, **p < 0.01, and ***p < 0.001.

The synergistic therapeutic schedule is shown in Figure 5A, the establishment of the tumor-bearing model was recorded as day −7, and the laser and US irradiation treatment was applied after 4 h of TiO_2-x_@GL injection on day 0. The subsequent US treatment was administered on the 3rd and 5th days. Ti element concentrations were significantly increased in tumor tissues after the intravenous injection of TiO_2−x_ NPs or TiO_2−x_@GL. The GL increased the accumulation of TiO_2-x_ NPs nearly 3-fold compared to that of the TiO_2−x_ group (Figure 5B). The body weight of the mice showed no significant change during the treatment among all groups (Figure 5C). The tumor weight in the TiO_2−x_@GL + laser + US group was decreased significantly (*p* < 0.01) compared to control and TiO_2−x_@GL groups, and the decrease was significant (*p* < 0.05) compared to those of TiO_2−x_@GL + laser and TiO_2−x_@GL + US groups, respectively on day 15 (Figure 5D). Meanwhile, the tumor volume exhibited no growth in the TiO_2−x_@GL + laser + US group (Figure 5E), and the synergistic therapy contributed to prolonged morbidityfree survival (Figure 5F). H&E staining showed obvious pathological changes in the TiO_2−x_@GL + laser + US group as the tumor cells swelled, and Tunel assay showed increased apoptosis in the TiO_2−x_@GL + laser + US group. The expression of Ki-67 was significantly down-regulated in xenograft tumors after TiO_2−x_@GL + laser + US treatment. These data show that TiO_2−x_@GL + laser + US suppressed tumor growth *in vivo*.

### Safety evaluation of TiO_2-x_@GL

**FIGURE 6:**
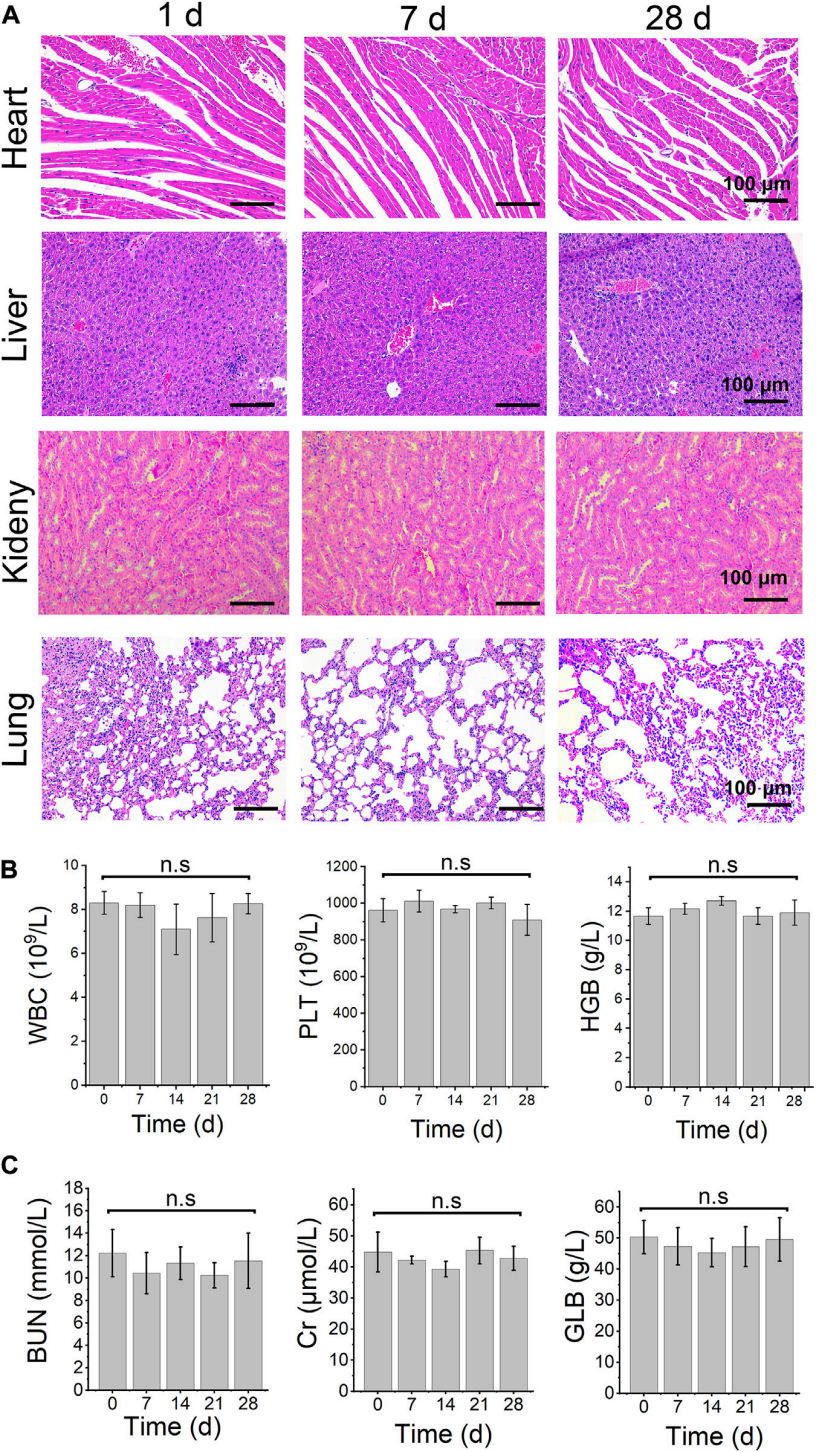
safety evaluation. **(A)** H&E staining images in main organ tissues of TiO_2−x_@GL combined with laser and US irradiation on days 1, 7, and 28. Blood routine examination **(B)** and biochemical parameters **(C)** of TiO_2−x_@GL combined with laser and US therapy from day 0 to day 28. WBC: white blood cell; PLT: platelet; HGB: Hemoglobin; BUN: urea nitrogen; Cr: Cr-reactive protein; GLB: Globulin.

The safety evaluation of TiO_2-x_@GL was revealed by the pathological changes of important organs and key blood and biochemicial parameters. H&E staining showed that the heart, liver, kidney, and lung showed no obvious changes on days 1, 7, and 28 (Figure 6A). The blood markers, including white blood cells (WBC), platelets (PLT), and hemoglobin (HGB) showed no significant change among different groups. Meanwhile, the biochemicial parameters, including urea nitrogen (BUN), C-reactive protein (Cr), and globulin (GLB) did not change compared to the control group (Figure 6B).

## Discussion

As a traditional nanomaterial, titanium dioxide nanoparticles (TiO_2_ NPs) have been extensively applied in the field of anti-bacteria (Esteban Florez et al., 2018), particularly in cancer therapy (Rodriguez-Barajas et al., 2022), owing to their unique characteristics of low toxicity, remarkable biological compatibility, and SDT and PDT activity. TiO_2_ NPs is a semiconductor material, the energy gap of the anatase type is 3.23 eV, and that of the rutile type 3.06 eV (Nosaka and Nosaka, 2016). The molecules of TiO_2_ NPs would go into an excited state upon absorbing a photon with energy above or equal to the gap energy, and created negative electrons (e-) in the conduction, leaving a positive charged hole (h+) (George et al., 2011). This allowed them to induce reactive oxygen species (ROS), which was the key factor used by the researchers to kill cancer cells in other cancer therapy studies (Ganji et al., 2022).

Studies indicated that TiO_2_ NPs have relatively low efficiency with respect to inducing ROS, which is mostly stimulated by UV light (Wang et al., 2022), which is harmful to cells to a certain degree (Haney et al., 2022). To improve the efficiency of TiO_2_ NPs in ROS induction, Han et al. (2018) used the aluminum (Al) formed an oxygen-deficient TiO_2-x_ layer on the surface of the TiO_2_ nanocrystals, which promoted and enhanced the separation of e- and h+ from the band structure under ultrasound irradiation, and significantly improved the effect of sonodynamic therapy on tumors. On this basis, we designed the TiO_2-x_ to enhance their ability to generate ROS, and tumor therapy efficacy. Our results showed that TiO_2-x_ was nearly 50 nm with the spherical morphology and 100 nm of DLS diameter, and the characteristic results were consistent with those of a previous study, indicating that synthesis was successful and is suitable for subsequent research (Li et al., 2022).

The application of TiO_2_ NPs for PTT and SDT on cancer treatment had unique advantages and obvious disadvantages. This technology requires high concentrations of TiO_2_ NPs and sufficient duration of illumination, and exhibits low target-killing ability, making it a focal point in numerous studies on drug delivery systems (Zhang et al., 2012; Torres-Romero et al., 2020). To enhance the targeting ability and stimulation of nanoparticles in cancer tissues, we used the GL as the guideline. Previous studies showed that the tumor cells would actively uptake GL for their growth. Despite being an abundant amino acid, GL is lacking in the tumor-bearing body (Chen et al., 1990), and the exogenous GL supplementation is preferentially transported to tumor tissues. Further, the tumor-bearing model study indicated that GL supplementation would not stimulate tumor growth (Fahr et al., 1994), indicating that the addition of GL is safe for tumor-bearing individuals. Based on the above studies, we designed a TiO_2-x_ (TiO_2-x_@GL) coated with GL to achieve this goal. Our results indicated that TiO_2-x_ was successfully embedded with GL, and with a hydrodiameter of approximately 100 nm, which is consistent with the findings of numerous previous studies (Kim et al., 2022; Salah et al., 2022). In addition, TiO_2-x_@GL can be activated at 1064 nm NIR-II bio-window, thus circumventing the potential challenge caused by UV illumination. Meanwhile, the concentration, US irradiation energy, and the light type and intensity for ROS generation by TiO_2-x_@GL is prior to many nanoparticles previously reported (Zhang and Sun, 2004; Chun-Hui et al., 2009).

SDT and PTT had different mechanisms in killing tumor cells, including ROS generation and high temperature. This design of TiO_2−x_ with oxygen defects in the crystalline structure had been demonstrated to improve the separation efficacy of electrons and holes and subsequently enhance photocatalytic efficacy. TiO_2−x_ also also proved to enhance the temperature in tumor atmosphere response under NIR irradiation in the NIR-II biowindow, which assist hyperthermia for killing tumor cells. Therefore, the TiO_2-x_@GL could inhibit tumor growth *via* inducing ROS under both US and laser irradiation, and enhance temperature to assist killing tumor cells after laser irradiation.

Despite the many advantages of nanoparticles in tumor treatment, it is difficult for them to penetrate barriers, particularly the tumor capsule. Studies indicate that only ≤1% nanomedicine doses can reach tumor tissues and exert their functions (Nakamura et al., 2016). Our results showed that with the help of GL, the concentration of TiO_2-x_@GL increased approximately three-fold in the tumor tissues, which significantly improved their killing efficacy.

Although the joint application of SDT and PTT has been considerably successful in tumor therapy, the sole application of either form of therapy is less efficient in inducing ROS. Therefore, recent studies mainly focused on the synergistic sonodynamic and photothermal treatment (Gao et al., 2019; Cao et al., 2022; Du et al., 2022). Tan used TiO_2_ NPs as the core to build a nano-sonosensitizer, combining it with the PD-1 checkpoint blockade therapy strategy, and found it effectively inhibited tumor cell proliferation and stopped tumor metastasis (Tan et al., 2021). In addition, Xue et al. designed a TIO_2_@PT/GOX (TPG)-mediated sonodynamic therapy (SDT) and starvation therapy (ST) that promotes systemic tumor suppression (Zhao et al., 2022). Therefore, in this study, we carried out a SDT/PTT synergistic strategy for evaluation.

Our results showed that the TiO_2-x_@GL + SDT + PTT yielded more optimized performance than the TiO_2-x_@GL + SDT/PTT. The joint application of the aforementioned treatments had no effects on the weights of the tumor-bearing mice, but significantly reduced the tumor weight and prolonged the duration of morbidity-free survival. Further mechanical research showed that combination treatment inhibited tumor cell proliferation, and triggered cell apoptosis to anticancer, which is consistent with the elevated ROS induced bio-effects (Wei et al., 2022). In addition, we found that TiO_2-x_ and TiO_2-x_@GL can inhibit cell migration, which is consistent with the results we previously found in studies onTiO_2_ NPs study (Mao et al., 2018), suggesting that TiO_2-x_@GL may have the ability to inhibit tumor metastasis and prolong survival time. To evaluate the safety of the combination treatment, we observed the pathological changes of main organs and the key biochemical parameters. All the results showed no significant change, indicating that TiO_2-x_@GL had low toxicity to normal cells similar to TiO_2_ NPs (Javed et al., 2022).

## Conclusion

Our study reported a GL coated oxygen-deficient TiO_2-x_ to enhance their sonocatalytic and photothermal efficiency in tumor therapy. The oxygen-deficient TiO_2-x_ structure enhanced the separation efficiency of e− and h+, and endowed the nanoparticle with high photothermal-conversion in the 1064 nm NIR-II biowindow. The GL shell guided the target delivery of nanoparticles and enhanced their accumulation in the cancer tissues, which improved their anticancer efficiency considerably. This study presents a nanomedicine and high efficacy of a combined treatment plan for cancer therapy, and proposes a strategy for designing an anticancer nanomedicine.

## Data Availability

The original contributions presented in the study are included in the article/supplementary material, further inquiries can be directed to the corresponding authors.
